# Quantifying defence cascade responses as indicators of pig affect and welfare using computer vision methods

**DOI:** 10.1038/s41598-020-65954-6

**Published:** 2020-06-02

**Authors:** Poppy Statham, Sion Hannuna, Samantha Jones, Neill Campbell, G. Robert Colborne, William J. Browne, Elizabeth S. Paul, Michael Mendl

**Affiliations:** 1 Animal Welfare and Behaviour Group, Bristol Veterinary School, University of Bristol, Langford House, Langford, BS40 5DU UK; 2 Department of Computer Science, University of Bristol, Merchant Venturers Building, Woodland Road, Bristol, BS8 1UB UK; 3 School of Veterinary Science, Massey University, Palmerston North, 4410 New Zealand; 4 School of Education and Centre for Multilevel Modelling, University of Bristol, 35 Berkeley Square, Bristol, BS8 1JA UK

**Keywords:** Emotion, Animal behaviour

## Abstract

Affective states are key determinants of animal welfare. Assessing such states under field conditions is thus an important goal in animal welfare science. The rapid Defence Cascade (DC) response (startle, freeze) to sudden unexpected stimuli is a potential indicator of animal affect; humans and rodents in negative affective states often show potentiated startle magnitude and freeze duration. To be a practical field welfare indicator, quick and easy measurement is necessary. Here we evaluate whether DC responses can be quantified in pigs using computer vision. 280 video clips of induced DC responses made by 12 pigs were analysed by eye to provide ‘ground truth’ measures of startle magnitude and freeze duration which were also estimated by (i) sparse feature tracking computer vision image analysis of 200 Hz video, (ii) load platform, (iii) Kinect depth camera, and (iv) Kinematic data. Image analysis data strongly predicted ground truth measures and were strongly positively correlated with these and all other estimates of DC responses. Characteristics of the DC-inducing stimulus, pig orientation relative to it, and ‘relaxed-tense’ pig behaviour prior to it moderated DC responses. Computer vision image analysis thus offers a practical approach to measuring pig DC responses, and potentially pig affect and welfare, under field conditions.

## Introduction

Objective measurement of animal welfare under field conditions is an important goal in animal welfare science. Progress has been particularly strong in on-farm welfare assessment which initially focused on cataloguing and quantifying the resources provided to animals (‘inputs’: e.g. trough space, drinker access, lying substrate, ventilation etc.^1^) and used this information to make inferences about welfare. More recently, attention has widened to include measurements of the animals themselves (‘outputs’: e.g. lameness, indicators of injury or disease, abnormal behaviour^2–4^) in order to get more direct evidence about their welfare, as in EU Welfare Quality on-farm welfare assessment protocols^5–7^.

Because concerns about animal welfare are, for many, based on an assumption that non-human animals can experience negative affective (emotional) states and therefore suffer^8–11^, there is a particular need to develop validated indicators of animal affect that can be implemented quickly and easily. To this end, a variety of behavioural measures have been developed for use under farm conditions (e.g. human approach tests, novelty tests, Qualitative Behavioural Assessment^12–15^). Some have been systematically validated against other indicators or by using manipulations of affective state grounded in a clearly-argued rationale, others less so^12,16^. In many cases, a major barrier to uptake is the time required to collect and interpret the relevant data^17–19^. Consequently, there is still a need for new validated and reliable indicators of animal affect and welfare that are easy to implement under field conditions.

One potentially promising measure is the ‘Defence Cascade’ (DC) response shown to sudden, unexpected stimuli^20–23^. The DC is an adaptive suite of responses evolved to ensure appropriate detection, evaluation and response to alerting stimuli, and conserved across species. It involves initial detection and immediate response (*startle*) to a stimulus such as an unexpected noise^20–22^, monitoring and evaluation of the stimulus accompanied by *freezing*/immobility^22,24^, and a final response of either defensive/escape behaviour or resumption of previous activities. Critically, components of the DC response are modulated by affective state in humans and rodents, making them potentially valuable indicators of these states.

The startle component, a chained series of rapid flexor reflex movements that cascade through the body and serve a protective function (e.g. eye blink), acts as a behavioural interrupt and enhances vigilance by switching attention towards potential threats^20,21,25^. In humans and rodents, negative states appear to potentiate startle magnitude. For the human eye blink, electromyography is usually used to quantify the strength of contraction of the orbicularis oculi muscle that closes the eye. For rodents, the magnitude of movements such as jumping are recorded using a load platform. For example, humans show larger startle responses when viewing or anticipating unpleasant pictures^26–30^ or negative emotional expressions^31^, anticipating shock^32^, in the presence of odours previously paired with a stressful social experience^33^, and even when exposed to sweat odours from anxious donors^34^. In rodents, many studies demonstrate potentiated startle in subjects exposed to conditioned stimuli predicting an aversive event^25,28,35–38^, an effect that may be mediated by corticotropin-releasing hormone^39^ and attenuated by anxiolytic drugs (e.g. diazepam^37^). Startle-potentiating effects of aversive conditioned stimuli are also observed in monkeys^40^.

Conversely, startle magnitude is often attenuated in people exposed to pleasant pictures^27,29^, sounds^41^, or conditioned visual stimuli paired with the removal of a painful stimulus and hence ‘relief’^42^, and in rodents exposed to conditioned visual or olfactory stimuli associated with rewards such as food^43–46^ and rewarding brain stimulation^47^.

The links between negative states and startle potentiation and positive states and startle attenuation are not always observed. For example, startle magnitude sometimes increases in people anticipating pleasurable pictures^48,49^ or monetary reward^32,50^ (although startle magnitude attenuates *following* monetary reward^50^). Bach^51^ provides a theoretical and computational model which reconciles these findings with those described previously by assuming that forgone opportunities due to a brief startle response are lower than those due to being injured (e.g. by a predator) if one does not startle. The model thus predicts an increased startle, resulting in lower overall costs, when beneficial opportunities are available such as during reward anticipation. Other exceptions include that startle was potentiated when viewing the names of loved ones compared to neutral or famous names^52^ and that anxiolytics had no effects on fear-potentiated startle^53^. Likewise, enrichment^25^ and preferred home cage odour^54^ did not attenuate startle in rats, and chronic mild stress failed to potentiate startle in mice^55^.

The freeze component of the DC response may function to allow effective processing of new information about potential danger, and to decrease the likelihood of detection by predators^22,56,57^. Negative affective states associated with increased anticipation of negative events^58^ may thus favour prolonged freezing responses that minimise chances of being detected by a predator and reflect a higher threshold of evidence required to judge that the environment is safe. For example, freezing in rodent aversive conditioning studies is frequently used as an indicator of a fear-like state^59–61^ and is potentiated by induced stress^62^ and attenuated by drugs and other treatments assumed to generate a less negative state^63–67^. Rats selected for an anxiety and depression-like phenotype show higher levels of freezing in aversive conditioning tests^68^ and dogs freeze for longer in response to negatively than positively valenced stimuli^69^. As with startle, there are exceptions to these findings such as the enhancement of unconditioned freezing in appetitive contexts^70^ and the failure of GABA agonists, assumed to have an anxiolytic function, to modify freezing in mice^71^.

Variation in startle and freeze components of the DC response thus offer potential as new indicators of affective valence and hence welfare. Such variation is likely to reflect a combination of influences on an animal’s current affective state, including temperamental and environmental factors, and hence can provide a summary readout of how particular individuals with particular experiences and genetic predispositions are being affected by their current situation. Preliminary studies of startle responses have been carried out in some farm animal species (e.g. pigs^72–74^; cattle^75^; sheep^76^), and a recent study by Ross *et al*.^77^ demonstrated attenuated startle responses in hens living with, relative to without, preferred enrichments. However, a major challenge to the use of DC responses as indicators of affective state and welfare in the field is quick and accurate measurement of these rapid and subtle behavioural patterns. ‘Gold standard’ measures such as direct behavioural observations (e.g. detailed by-eye video analysis) may be manageable for individuals or small experimental groups under lab conditions, but are slow and/or impractical in the field. One possible solution to this challenge is to use computer vision techniques to implement real-time automated analysis of video-recorded DC responses^78,79^. Successful development of this approach would allow cheap, objective and rapid measurement of DC responses on farm, or in other contexts such as abattoirs, using just a video camera, a standardised eliciting stimulus, and the required software.

Here we explore this possibility in a commercially important farm animal – the pig – by evaluating the validity of computer vision image analysis against ‘ground truth’ data provided by human behavioural observation which has been used successfully in previous studies of pig DC behaviour^72–74^. We also collect force, kinematic, and depth-camera (Kinect) measures for comparison, as these are widely used to assess movement in other species. Finally, we evaluate whether contextual factors influence the DC response. We choose the pig because it shows characteristic DC behaviour when unexpectedly disturbed, usually involving a whole-body startle and movement to a tense standing position, sometimes accompanied by a bark vocalization^72^, followed by a period of immobility or freezing during which the animal appears to be monitoring or attempting to detect the source of the disturbance. These responses terminate when the pig flees or resumes ongoing behaviour. Successful development of an automated measure of DC responses will open the way for validating variation in startle and freeze behaviour as an indicator of affective valence in pigs and trialling the approach in groups of animals and on farm^73,80^.

## Materials and Methods

### Animals and housing

Twelve pigs (Large White x Landrace) were sourced from a commercial farm at approximately seven weeks of age. They were housed in two straw-bedded rooms (4.6 m × 4.6 m), naturally lit and supplemented with artificial light between 0700 and 1900, with a target temperature of 20 °C, and each holding three males and three females. Pigs were fed to appetite twice daily and water was provided ad libitum. They were weighed once a week. Studies were ethically reviewed by the Bristol University Animal Welfare and Ethics Review Body and carried out in accordance with the Animals (Scientific Procedures) Act 1986 (Project Licence: PPL 30/2867).

### Test room and equipment

The Test room contained a force-measuring pen, custom-built by Solutions for Research (https://www.solutionsforresearch.co.uk/) and consisting of a 1.31 ×1.33 m load platform fitted with four load cells surrounded by 1 m high walls, three of which were made of 2” wire mesh whilst the fourth had a clear polycarbonate guillotine door through which pigs could enter. Fast capture video cameras (3 monochrome Point Grey Dragonfly Express cameras running at 200 Hz) were positioned on two sides of the force-measuring pen and overhead, with the overhead position normalised by considering the location of the four corners of the pen. A standard video camera was set up for filming from one side of the pen. A Microsoft Kinect v1 camera running at 30 Hz and collecting RGB and depth data was also positioned above the pen. During weeks 4 and 12 of the study, Kinematic data were collected using four infrared Qualisys cameras (ProReflex MCU240, Qualisys AB, Goteborg, Sweden) running at 200 Hz and positioned at the four corners of the pen. The set-up is shown in Fig. S1 in the Supplementary Information which also contains information on data capture and synchronisation.

### Habituation to test room

On arrival (week 1 of the study) pigs were given individual ear tags and then left undisturbed (except for cleaning and feeding) for three days. During weeks 1–2 the pigs were gradually habituated to human contact, moving to the Test Room and spending time on the load platform. They were initially introduced to the room in a group of six and food treats were available throughout. Over a period of days, the group sizes were reduced and time periods increased until each pig was spending at least 5 minutes alone on the platform.

### Testing

The aim of this study was to generate recordings of a large number of Defence Cascade events and then, using all these events, to compare metrics generated by different measurement techniques in order to establish the validity of computer vision measures of the DC response. To this end pigs were tested repeatedly to generate the dataset of recordings. Testing was carried out when the pigs were relatively young and light (weeks 3–5 of the study; 9–11 weeks of age; 20–40 kg) and subsequently when they approached slaughter weight (weeks 11–13 of the study; 17–19 weeks of age; 50–80 kg). In each phase, each pig received three Standard test sessions and one Kinematic test session. One pig had to be excluded from two sessions due to illness, thus giving 94 sessions in total.

During each Standard test session each pig was taken from its home pen to the Test Room and encouraged to enter the force-measuring pen with food treats. It was then given c.2.5 min to settle before the first test. During this time, the pig’s behaviour was observed live by PS and a ‘relaxed-tense’ score given using the rating scale shown in Table S1. A startling stimulus was then presented and the pig’s response was recorded for 30 s using the load platform, Kinect, fast-capture, and standard video cameras. Sessions comprised a maximum of 5 tests (stimulus presentations), each separated by c.5 min, after which the pig returned to its home pen.

During sessions 1 and 2, the startling stimulus was a remotely-activated bursting balloon next to the force pen. Pigs rapidly habituated to this stimulus and therefore, given the repeated testing required for this experiment in order to yield the dataset required for analysis (see above), it was deemed necessary to vary the stimulus for the remaining sessions to generate sufficient startle/freeze responses for a comparison of automated image analysis and ground-truth measures of these. In session 3 the bursting balloon was combined with a person either calling out or stepping out from behind a curtain. From session 4 onwards the stimulus was varied in each test, the stimuli used included; an unfamiliar researcher stepping out from behind a curtain, a bucket containing metal nuts being dropped, a model person being hoisted above the pig, an umbrella being opened towards them or a bin bag being waved.

During Kinematic test sessions in weeks 4 and 12, pigs were taken from their home pen to a small room where 10 mm passive reflective spherical markers were attached to their backs using double sided sticky tape. One was placed between the ears and three more were spaced down the spine. The final two markers were transverse to each other in the region of the sacroiliac joints; these identified the rear of the pig. Once markers were securely in place the pig was taken to the Test room and the test procedure continued in the same way as for Standard tests with all measures recorded as usual, plus kinematic data recorded for 15 s following each startling stimulus.

In total, 285 tests nested within 94 test sessions (each comprising up to 5 stimulus presentations) were carried out across both phases. Technical issues with some tests mean the final dataset for analysis was 280 tests.

### Observations of behaviour from video recordings

Recordings from the standard video camera were coded by-eye using the Observer program^81^. Each video was examined in slow motion, multiple times to record the behaviour exhibited in response to the stimulus (Jump and fall, Jump away, Jump on spot, Spin to face, Side step, Head up, Head turn, Muscle ripple, Ear prick, Sniff at/approach stimulus, Freeze; see Table S2). Coding continued until an outcome behaviour (Flee, Slowly leave, Return to normal behaviour; see Table S2) occurred which always happened within 30 s of the startling stimulus. An *Observer Startle Magnitude* score of 0–4 (least to most intense) was generated from the coded behaviour according to a rating scale described in Table 1. A total *Observer Freeze Duration* (s) was also calculated. Any freezes that lasted less than a second were reviewed and those less than 0.4 s were re-classified as ‘no freeze’ because they did not show the tension/immobility required by our behavioural definition: ‘muscles tensed, whole body stationary, ears often pricked’.

**Table 1:** Classification of behaviour used to create the Observer Startle Magnitude Score.

Startle Magnitude Score	Strongest Reaction Seen	Behaviour Description
4	Flee	Rapid exit from the force pen in response to stimulus
3	Large jump with movement, typically away from the stimulus	Jump and fall (jump in the air in response to stimulus, knees or abdomen touch the ground on landing) Jump away (Jump in response to stimulus, and away from it)
2	Jump on spot or spin around to face stimulus	Jump on spot (Jump in response to stimulus and land standing in same location) Spin to face (Whole body movement to re-orientate towards stimulus)
1	No jump, but reaction (Side step, Head up, Head turn, Muscle ripple, Ear prick)	Side step (One sideways movement with one or two feet, typically away from the stimulus) Head up (Lift head suddenly in response to stimulus, snout no longer in close contact with floor) Head turn (Head turn in the direction of the stimulus) Muscle ripple (Tensing of muscles or muscle twitch in response to stimulus) Ear prick (Ears pulled back or pointing upwards in response to stimulus)
0	No startle reaction	Pig’s behaviour did not change in response to the stimulus

### Image analysis (IA) data

All IA measures were derived from the overhead fast capture camera (200 Hz frame rate) as it gave the clearest unobstructed view of the pig. A view from above is also likely to avoid obstructions such as pen walls, posts and other pigs under field conditions. To measure the magnitude of the startle response to the stimulus, accelerations of highly textured image regions were estimated using sparse feature tracking. For freeze durations, both speed and acceleration were extracted. Sparse feature tracking considers only the easiest regions to track and hence improves accuracy compared to dense feature tracking^82^. Birchfield’s implementation of the Kanade Lucas Tomasi tracker was utilised (KLT; https://cecas.clemson.edu/~stb/klt/), and tracked points are referred to as KLT points. Justification for using points rather than centroids is provided in the Supplementary Information.

The magnitude of the initial response to the startling stimulus (*KLT Acceleration Startle Magnitude*; acceleration being closely related to force) was defined as the maximum acceleration of 50 KLT points (pixels/frame^2^) in a temporal window 0.7 s after the startle stimulus (see Supplementary Information). The tracker was always initialized with 150 points. If points were lost by the tracker, new ones were initialized. For our calculations, we only considered the 50 points with the highest acceleration that were successfully tracked for 3 consecutive frames. *KLT Speed Freeze Duration* was defined as the total time the speed of the 50 fastest KLT points (pixels/frame) was below an empirically determined threshold for a continuous period of at least 0.4 seconds (see above). Thresholds were calculated using 10-fold cross validation with 90% training and 10% testing data repeated 10 times for all data (see Supplementary Information). The same method was used to determine *KLT Acceleration Freeze Duration*, in case acceleration of the KLT points provided a better predictor of freeze duration than speed of the KLT points.

### Load platform data

The four load cells of the platform each generated an analogue signal encoding change in force into change in voltage. This was captured at a rate of 400 Hz, converted into digital form and stored on computer to generate a time series synchronised with the other data and used in all subsequent analyses (see Supplementary Information). Signals from the four transducers were summed to give a measure of total instantaneous vertical force and normalised by the mass of each pig as this changed significantly over the course of the experiment and heavy pigs exert more force than lighter ones. The weight of the pig was determined from the average total force reading prior to the startle stimulus. All readings had this weight subtracted and were subsequently divided by this value to get vertical acceleration in units of gravities, *g*, where one *g* is 9.8 m/s^2^. *Load Platform Startle Magnitudes* were calculated as the peak absolute acceleration during a 0.7 s window after the startle stimulus. *Load Platform Freeze Durations* were calculated using load platform acceleration measures and thresholding in the same way as for Image Analysis (IA) data.

### Kinect data

Depth maps were extracted from Kinect data collected at a 30 Hz frame rate (see Supplementary Information). For each depth map sequence, a time series recording the vertical displacement of the pigs’ centroids for the total duration of each test was constructed. These time series were used in all subsequent analyses. Vertical centroid speed was the magnitude of the first differential with respect to time in these series. Acceleration magnitude was the second differential. Startle magnitudes and freeze durations were extracted using both of these measures. *Kinect Speed* *Startle Magnitude* and *Kinect* *Acceleration Startle Magnitude* were calculated as, respectively, the peak absolute values of the vertical centre of mass velocity (mm/frame), and acceleration (mm/frame^2^) during a 0.7 s window following the startle. The method used to calculate *Kinect Speed Freeze Duration* and *Kinect* *Acceleration Freeze Duration* was the same as that used for the IA data.

### Kinematic data

Kinematic data were recorded for 15 s following the startle stimulus and sub-sampled at 30 Hz. We extracted the magnitude of velocities and accelerations for each spherical marker on the pig individually and then took the average of the magnitude of those markers’ trajectories. We only considered motion in the vertical direction as this was most likely to correspond to load platform readings. *Kinematic Velocity Startle Magnitude and Kinect Acceleration Startle Magnitude* were, respectively, the peak absolute values of vertical velocity (mm/frame) and acceleration (mm/frame^2^) during a 0.7 s window after the startle stimulus. Freeze durations were not calculated due to the short 15 s recording window which failed to capture longer duration freezes.

### Statistical analysis

Data extraction generated the following variables. *Startle Magnitude estimates*: Observer Startle Magnitude Score; KLT Acceleration Startle Magnitude; Kinect Acceleration Startle Magnitude; Kinect Speed Startle Magnitude; Kinematic Acceleration Startle Magnitude; Kinematic Velocity Startle Magnitude; Load Platform Startle Magnitude. *Freeze Duration estimates*: Observer Freeze Duration; KLT Acceleration Freeze Duration; KLT Speed Freeze Duration; Kinect Acceleration Freeze Duration; Kinect Speed Freeze Duration; Load Platform Freeze Duration.

#### Comparisons of automated readouts with observer ground truth measures – startle magnitude

Due to inequality of variances and a highly skewed distribution of Load Platform data which was resistant to transformation, non-parametric statistics were used. We compared ground truth Observer Startle Magnitude scores to other measures of startle magnitude using Spearman Rank correlations. Given the hierarchical nature of the data, data points were not independent rendering derived p-values inaccurate. We therefore constructed multilevel regression models in MLwiN^83^ with Test (n = 280) nested within Session (n = 8) and Session nested within Pig (n = 12). The response variable was Observer Startle Magnitude score (0–4), thus requiring use of an ordinal response multinomial model, with a reference category of 4 (Flee). In this model, negative estimated coefficients of predictor variables would therefore indicate a positive relationship with Observer Startle Magnitude. We carried out univariate analyses by adding each of the other measures of startle magnitude individually into the model as predictors of our response variable and used Wald tests to examine the significance of the term in the model and thus generate approximate p-values. The predictors were KLT, Kinect, Kinematic and Load Platform Acceleration, and Kinect and Kinematic Velocity estimates of startle magnitude.

#### Comparisons of automated readouts with observer ground truth measures – freeze duration

Due to the large number of zero values present in this dataset, Spearman Rank correlations were again used to compare the ground truth Observer Freeze Duration with other measures. A multilevel model was constructed in MLwiN to derive p-values that accounted for the nested structure of the data, with Observer Freeze Duration as the response variable. Although the data were not amenable to being transformed to normality, we used a normal rather than binary model to allow comparison to the correlation results. In this model, a positive estimated coefficient of a predictor variable indicated a positive relationship with the response variable. The predictors were Kinect, KLT and Load Platform Acceleration, Kinect Speed, and KLT Speed estimates of freeze duration. To check that significant relationships were not solely influenced by the large number of zero values, we calculated the sensitivity, specificity and positive/negative predictive values of each of the freeze or no-freeze categories. Finally, for robustness we excluded the zero values altogether and examined the correlation coefficients for those cases where both measures scored a freeze as occurring.

#### Analysis of the effects of other factors on observer measures of startle and freeze

Multilevel models were used to investigate the possible effects of a range of factors (e.g. pig sex, weight, behaviour prior to test, orientation in apparatus, time of day, experience of test across time and within a day, startle stimulus, test session type; Table S3) on observer measures of startle and freeze responses. As above, an ordinal response multinomial model was used for startle magnitude data with Observer Startle Magnitude (0–4) as the response variable. However for freeze data, where the comparison to continuous measures required in the above analyses was now not essential, we converted the non-normal data into a binary freeze/no-freeze variable and used a binomial model with a logit link function. The hierarchical structure of the data was as described previously.

Univariable analysis was first completed by adding each of the possible influencing factors individually into the multilevel model and recording whether they were significantly related to the response variable. A multivariable model was then constructed by sequentially adding in each of the significant influencing factors (most significant first) and checking at each stage that all previously added factors in the model remained significant. Once this had been done, any non-significant factors were entered into the model one by one. If they were now significant, the model-building process was repeated until none of the remaining factors, when introduced into the model, were found to be significant. Wald tests were then used to check whether the levels of hierarchical structure within the model were significant. If not, they were removed from the final model.

## Results

### Comparisons of automated readouts with observer ground truth measures – startle magnitude

**Figure 1:**
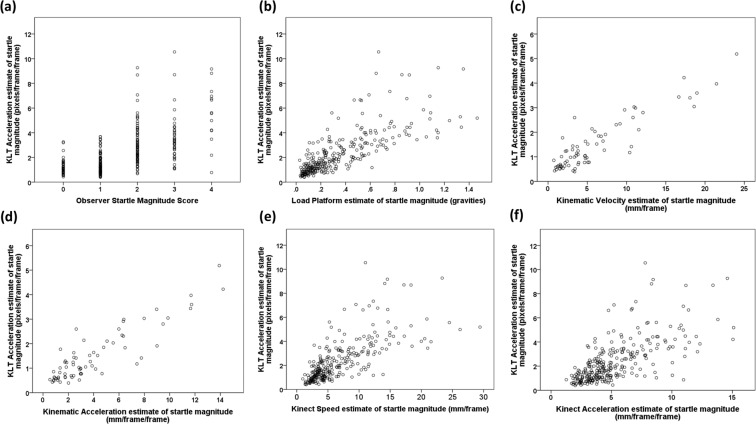
Scattergrams of the relationship between KLT Acceleration estimates of startle magnitudes (pixels/frame^2^) and those provided by (**a**) Observer Startle Magnitudes scores; and (**b**) Load Platform (gravities); (**c**) Kinematic Velocity (mm/frame); (**d**) Kinematic Acceleration (mm/frame^2^); (**e**) Kinect Speed (mm/frame); (**f**) Kinect Acceleration estimates (mm/frame^2^).

The ground truth Observer Startle Magnitude measure was significantly positively correlated with all automated measures of startle magnitude (Table 2). The strongest correlation was with the Kinematic measures, although these were only available for a subset of the data. Multilevel models showed that the Load Platform and then KLT Acceleration measures were the strongest predictors of the Observer Startle Magnitude scores. (Table 2). KLT Acceleration data were strongly positively correlated not just with Observer Startle Magnitude, but also with all the automated measures, which are widely used to measure movement (Load Platform, r_s_ = 0.824: n = 280, P < 0.001; Kinematic Velocity: r_s_ = 0.821, n = 70, P < 0.001; Kinematic Acceleration: r_s_ = 0.816, n = 70, P < 0.001; Kinect Speed: r_s_ = 0.772, n = 280, P < 0.001; Kinect Acceleration: r_s_ = 0.693, n = 280, P < 0.001; Fig. 1).

**Table 2:** Relationships between the Observer Startle Magnitude score and the Kinect, Kinematic, Load Platform and KLT estimates of startle magnitude.

Measure	n	Corr. Coef. (p-value)	Coefficient estimate (with SE)	Wald Χ^2^ (p-value)
Kinect Acceleration	280	0.529 (<0.001)	−0.395 (0.047)	70.126 (<0.001)
Kinect Speed	280	0.606 (<0.001)	−0.243 (0.028)	75.447 (<0.001)
Kinematic Acceleration	70	0.743 (<0.001)	−1.013 (0.166)	37.103 (<0.001)
Kinematic Velocity	70	0.729 (<0.001)	−0.519 (0.088)	34.557 (<0.001)
Load Platform	280	0.706 (<0.001)	−5.565 (0.524)	112.978 (<0.001)
KLT Acceleration	280	0.654 (<0.001)	−0.847 (0.088)	92.855 (<0.001)

Correlation Coefficients and associated p-values were calculated using a Spearman Rank Correlation. The Coefficient Estimate, related Standard Error, Wald statistic and associated p-value were from the ordered multinomial multilevel models.

### Comparisons of automated readouts with observer ground truth measures – freeze duration

The ground truth Observer Freeze Duration measure was significantly and positively correlated with the Kinect, Load Platform and KLT measures. Multilevel models showed that the Kinect Speed and then KLT Speed measures were the strongest predictors of the Observer Freeze Duration scores. (Table 3).

**Table 3:** Relationships between the Observer Freeze Duration measure and the Kinect, Load Platform and KLT estimates of freeze duration.

Measure	n	Corr. Coef. (p-value)	Coefficient estimate (with SE)	Wald Χ^2^ (p-value)
Kinect Acceleration	282	0.837 (<0.001)	0.876 (0.030)	381.299 (<0.001)
Kinect Speed	282	0.833 (<0.001)	0.867 (0.027)	422.209 (<0.001)
Load Platform	283	0.784 (<0.001)	1.030 (0.0.34)	397.375 (<0.001)
KLT Acceleration	283	0.771 (<0.001)	0.908 (0.034)	351.285 (<0.001)
KLT Speed	283	0.821 (<0.001)	0.848 (0.027)	412.752 (<0.001)

Correlation Coefficients and associated p-values were calculated using a Spearman Rank Correlation. The Coefficient Estimate, related Standard Error, Wald statistic and associated p-value were from the multilevel models.

**Figure 2:**
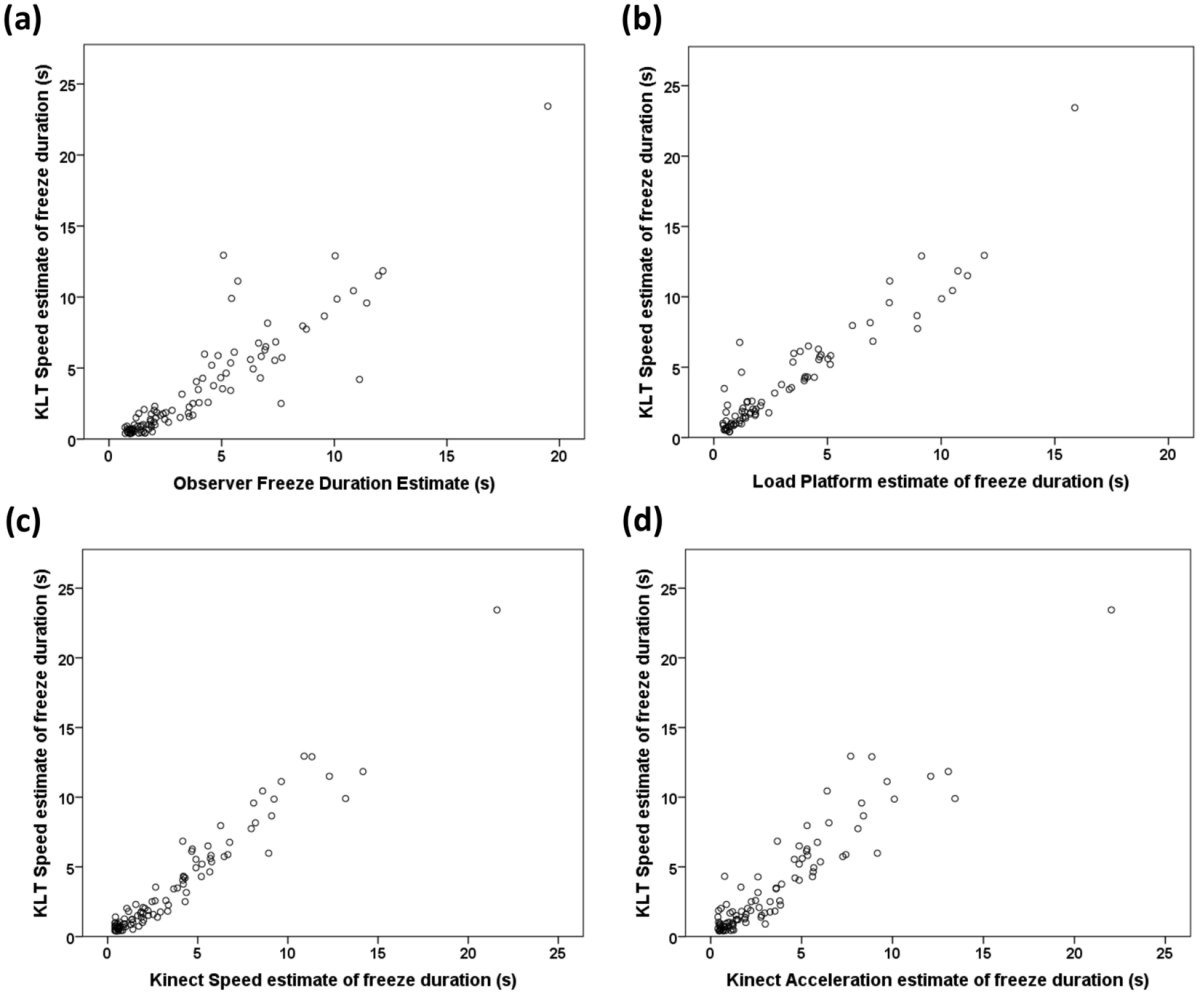
Scattergrams of the relationship between KLT Speed estimates of freeze durations and those provided by (**a**) Observer; (**b**) Load Platform; (**c**) Kinect Speed; (**d**) Kinect Acceleration estimates. Estimation of freeze durations (all units are seconds) by each method is explained in the Methods.

Given the large number of Observer Freeze Duration zero values, we investigated how well the different measures predicted whether an Observer Freeze was detected or not (Table 4). Sensitivities were variable, with the Kinect measures being most sensitive (0.76–0.80), followed by KLT Speed (0.71) and then the Load Platform (0.59) and KLT Acceleration (0.55) measures. The same order was evident for Negative Predictive Value which varied from 0.80 to 0.68. Specificities and Positive Predictive Values were all greater than 0.9 apart from those of the Kinect Acceleration measure (0.89 and 0.88 respectively). Correlation coefficients for the sample of True Positives (i.e. where both the Observer and the other measure scored a freeze as occurring) indicated that all measures were strongly positively correlated with Observer Freeze Duration (KLT Speed: r_s_ = 0.920, n = 104, P < 0.001; KLT Acceleration: r_s_ = 0.855, n = 79, P < 0.001; Kinect Speed: r_s_ = 0.889, n = 109, P < 0.001; Kinect Acceleration: r_s_ = 0.867, n = 114, P < 0.001; Load Platform: r_s_ = 0.877, n = 84, P < 0.001). Furthermore, KLT Speed estimates of freeze duration were strongly positively correlated with those derived from the other automated measures, when both measures detected a freeze (Kinect Speed: r_s_ = 0.938, n = 96, P < 0.001; Kinect Acceleration: r_s_ = 0.879, n = 98, P < 0.001; Load Platform: r_s_ = 0.894, n = 80, P < 0.001 Fig. 2). We did not calculate corresponding KLT Acceleration correlations due to the low sensitivity of this measure.

**Table 4:** Sensitivity, specificity, positive and negative predictive values of each automated measure as a predictor of whether a freeze was scored as occurring or not by a human observer.

Measure	True Positives	False Positives	True Negatives	False Negatives	Sensitivity	Specificity	Positive Predictive Value	Negative Predictive Value
Kinect Acceleration	114	15	122	29	0.797	0.891	0.884	0.808
Kinect Speed	109	11	126	34	0.762	0.920	0.908	0.788
Load Platform	84	4	133	59	0.587	0.971	0.955	0.693
KLT Acceleration	79	2	135	64	0.552	0.985	0.975	0.678
KLT Speed	102	7	130	41	0.713	0.949	0.936	0.760

Overall, computer vision KLT Acceleration estimates of startle magnitude, and KLT Speed estimates of freeze duration, correlated well with the corresponding Observer ground truth measures and with Load Platform, Kinematic and Kinect measures of pig movement.

### Effects of other factors on the observer measure of startle magnitude

The multilevel ordinal regression model of the influence of factors on Observer Startle Magnitude scores initially included Tests nested within Sessions within Pig. However neither of the sets of random effects for Session or Pig were significant when the predictor variables were added, and therefore the final model did not require any random effects. Factors significant in the final model are shown in Table 5. The primary reason that Session was significant is due to stronger startle magnitudes occurring in Session 4, when the startling stimuli started to be varied, compared to Sessions 5–8. The use of new stimuli resulted in increased startle magnitudes in comparison to the original balloon-burst stimulus, in particular when the bin bag was used. When compared to pigs that were rated as being calm prior to presentation of the first startling stimulus (relaxed-tense score of 1), those that received a score of 2 or 3 displayed a stronger startle reaction, but this effect was not seen for those animals with scores of 4 and 5. Larger startle reactions were seen when the pig was orientated away from the stimulus.

**Table 5:** Significant factors in the multilevel ordinal regression model of Observer Startle Magnitude.

Factor	Ref Cat	Variable	Corr. Coeff. (SE)	Variable Wald X^2^ (p-value)	Factor Wald X^2^ (p-value)
Session	Session 4	Session 1	−0.659 (1.531)	0.185 (ns)	**21.678 (<0.005)**
		Session 2	−0.399 (1.540)	0.067 (ns)	
		Session 3	1.685 (1.105)	2.327 (ns)	
		**Session 5**	**1.257 (0.451)**	**7.756 (<0.01)**	
		**Session 6**	**1.405 (0.468)**	**8.989 (<0.005)**	
		**Session 7**	**1.709 (0.608)**	**7.893 (<0.005)**	
		**Session 8**	**2.205 (0.521)**	**17.930 (<0.001)**	
Stimuli	Balloon	**Balloon & other**	**−2.684 (1.236)**	**4.711 (<0.05)**	**22.540 (<0.001)**
		**Model Person**	**−2.938 (1.466)**	**4.017 (<0.05)**	
		**Umbrella**	**−4.073 (1.451)**	**7.876 (<0.01)**	
		**Real Person**	**−3.265 (1.458)**	**5.014 (<0.05)**	
		**Bucket**	**−2.968 (1.443)**	**4.230 (<0.05)**	
		**Bin Bag**	**−4.688 (1.557)**	**9.061 (<0.005)**	
‘Relaxed-tense’ score	1 (calm)	**2**	**−1.029 (0.324)**	**10.068 (<0.005)**	**19.219 (<0.001)**
		**3**	**−1.064 (0.390)**	**7.431 (<0.01)**	
		4	1.047 (0.926)	1.279 (ns)	
		5	1.020 (1.215)	0.705 (ns)	
Orientation	3 (facing)	**1 (back)**	**−0.672 (0.325)**	**4.263 (0.05)**	**14.827 (<0.001)**
		**2 (90 deg)**	**−1.038 (0.271)**	**14.642 (<0.001)**	

The reference category of the response variable was a magnitude of 4 (Flee) and thus a negative estimated coefficient of a predictor indicates a larger response, whereas a positive coefficient indicates a smaller response. Bold text indicates effects that are significant at p < 0.05.

### Effects of other factors on the observer measure of whether a freeze response occurred

The multilevel model initially included Tests nested within Sessions within Pig. However again neither Session or Pig random effects were significant once predictors were added and so the final model did not include a hierarchical structure. The likelihood of a freeze response decreased from Session 1 to 2 and increased significantly in Session 4 when the startling stimuli started to be varied. There was also an increased likelihood of a freeze response when the pig was facing away from the stimulus at the point of testing, and for pigs with a ‘relaxed-tense’ score of 2 or 3 compared to a score of 1 prior to the first startling stimulus. However, pigs with a score of 4 or 5 did not show this increased likelihood of freezing (Table 6).

**Table 6:** Significant factors in the multilevel binomial regression model of Observer Freeze Occurrence.

Factor	Ref Cat	Variable	Corr. Coeff. (SE)	Variable Wald X^2^ (p-value)	Factor Wald X^2^ (p-value)
Session	Session 1	**Session 2**	**−1.577 (0.624)**	**9.389 (<0.05)**	**26.013 (<0.001)**
		Session 3	−0.046 (0.592)	0.006 (ns)	
		**Session 4**	**1.428 (0.680)**	**4.416 (<0.05)**	
		Session 5	0.363 (0.626)	0.336 (ns)	
		Session 6	−0.594 (0.641)	0.861 (ns)	
		Session 7	−0.785 (0.628)	1.562 (ns)	
		Session 8	−0.535 (0.610)	0.768 (ns)	
‘Relaxed-tense’ score	1 (calm)	**2**	**1.224 (0.395)**	**9.616 (<0.005)**	**16.824 (<0.005)**
		**3**	**0.977 (0.461)**	**4.498 (<0.05)**	
		4	−2.091 (1.257)	2.757 (ns)	
		5	0.650 (1.408)	0.213 (ns)	
Orientation to stimulus	1 (back to)	**2 (side on)**	**−0.813 (0.384)**	**4.492 (<0.05)**	**8.735 (<0.05)**
		**3 (facing)**	**−1.172 (0.398)**	**8.695 (<0.005)**	

A positive estimated coefficient of a predictor indicates an increased probability of a freeze occurring, whereas a negative coefficient indicates a decreased probability. Bold text indicates effects that are significant at p < 0.05.

## Discussion

In this study, we investigated the validity of computer vision image analysis (IA) as a measure of startle magnitude and freeze duration Defence Cascade (DC) responses in pigs, because of its potential as a practical tool for assessing these responses under field conditions. We used behavioural observation of video as our ground truth measure of startle and freeze responses (see^72–74^) and also collected load platform, kinematic, and Kinect depth-camera measures because these have been used to assess movement in other species.

Our KLT Acceleration image analysis estimate of startle magnitude, and estimates from all the automated measurements we made, were significantly positively correlated with the observer behavioural observation data. Kinematic measures generated the highest correlation coefficient which is unsurprising given that they are designed to capture detailed 3D movements and were derived using four carefully positioned cameras to measure precise movement of markers on the pigs. The Load Platform produced the next strongest correlation, followed by the image analysis KLT Acceleration data, with the Kinect measure generating the weakest correlations. Load Platform and KLT Acceleration were the best predictors of Observer Startle Magnitude in our multilevel model. The relatively weak predictive power of kinematic data in these analyses likely reflects the smaller sample size for this measure. Our image analysis estimate of startle magnitude was also strongly positively correlated with the load platform, kinematic and depth-camera measures.

Our KLT Speed and Acceleration image analysis estimates of freeze duration were strongly positively correlated with the observer measure, as were Kinect Acceleration and Speed, and Load Platform data. Because there were a high number of zeroes (non-freezes) during tests, we carried out sensitivity and specificity analyses of whether or not a freeze was detected against the human observer ground truth. All measures had high specificity – they rarely detected a freeze when the observer did not detect one – with computer vision KLT measures performing particularly strongly. Their positive predictive values – how well a detected freeze actually indicates that a freeze did occur – were therefore high. However sensitivity was lower, particularly for Load Platform and KLT Acceleration data, which frequently did not detect freezes when the observer did and hence generated a high proportion of false negatives. In many such cases, the observer recorded short freezes (<2 s) in which the body appeared tensed but isolated parts (e.g. ears) were moving. This short duration and residual movement may have accounted for the failure of other measures to detect a freeze response.

For true positive data (when both the observer and automated measures detected a freeze), automated measures of freeze duration were strongly positively correlated with those made by the observer, with KLT Speed and Kinect Speed performing best. Furthermore, KLT Speed estimates of freeze duration were also strongly positively correlated with the other automated measures.

In addition to analysing the relationship between different measures of startle and freeze behaviour, we also investigated factors that may influence expression of these behaviours in pigs tested under laboratory conditions. In our study, repeat-testing of individuals was necessary to generate sufficient examples of DC responses to establish associations between different measures of these responses, whilst also following 3Rs recommendations to minimise animal use. However, repeat-testing resulted in pigs habituating to the original startling stimulus (bursting balloon), as has been found in previous studies of pigs^72,84^. Consequently, we varied the nature of the stimulus within test sessions, starting in session 4, and startle response magnitude increased after this session. Similarly, the likelihood of a freeze response initially decreased from session 1 to session 2 when the bursting balloon was used repeatedly, but increased in session 4 when stimuli started to be varied within sessions. The type of stimulus used also affected DC responses with bin bag and umbrella stimuli, both involving rapid movement, being particularly effective at generating greater startle magnitudes than the balloon. Use of stimuli with a pronounced visual component may thus be more potent inducers of DC responses in pigs compared to purely auditory stimuli.

Larger startle magnitudes were observed when pigs were orientated away from the stimulus possibly because startling stimuli presented in this context induced a greater surprise reaction^20–22^. An alternative explanation is that those pigs orientated away from the stimulus were more likely to make a large jump with movement (Observer Startle Magnitude score of 3) in order to face the stimulus, whilst pigs already facing the stimulus may have been more likely to jump on the spot (score of 2). However, our finding that there was also an increased probability of freezing when pigs were orientated away from the stimulus lends support to the idea that the stimulus was indeed more startling and surprising in this context, including leading to increased post-startle processing (during freeze) to resolve the source of the event^21,22^.

A subjective rating of how ‘relaxed or tense’ the pigs were before the first stimulus was delivered during a test also influenced DC responses. For both startle magnitude and freeze occurrence an increase in reaction was seen for pigs scored as 2 or 3 when compared to the calmest pigs (scored as 1). However, this difference was not observed in pigs scored as 4 or 5 (least calm). In fact, the probability of a freeze reaction was decreased in pigs with a score of 4 compared to those with a score of 1. Although this seems counterintuitive, we observed informally that the least calm pigs appeared to pay least attention to their surroundings and were often attempting to leave the test pen. Consequently, delivery of the startling stimulus did not intrude into their already active behaviour. This re-emphasises the need for pigs to be settled and calm at the point of testing, for example in a home pen.

It is interesting to note that our models found corresponding effects of moderating factors on both startle magnitude and probability of freeze occurrence. Both increased when startling stimuli were altered, when the pig was facing away from them, and in pigs who were slightly or moderately tense prior to their presentation, although not for animals rated as tense or very tense who appeared to be focused on leaving the test pen. This coherence of effects indicates that startle magnitude and freeze probability may reflect a similar underlying construct, for example affective valence^25,28,38,40^.

In summary, we found that computer vision image analysis measures were comparable with force, kinematic and depth measurements at estimating ground truth observer measures of both startle magnitude and freeze duration/occurrence. These findings demonstrate that computerised image analysis of video recordings can be used to detect and quantify startle and freeze Defence Cascade responses in individual pigs, and hence has potential as a practical automated measure of these behaviours under field conditions. We also identified factors including startling stimulus characteristics, orientation relative to the stimulus, and ‘relaxed-tense’ behaviour that can moderate DC responses and hence should be borne in mind during further investigation of this behaviour. The similarity of these moderating effects across both startle and freeze behaviours indicates that these two forms of DC response may represent similar underlying constructs such as affective valence. If variation in startle and freeze behaviour does indeed reflect affective state in pigs, as it appears to in other species^20–22,28,59–61^, computer vision image analysis may thus provide a novel method for assessing pig welfare in the field. Further research on the link between affective state and pig startle and freeze behaviour, the capacity of image analysis to measure these behaviours in groups of pigs, and the utility of the approach under field conditions, including startle stimulus design and issues of habituation, is needed to realise this potential^73,78,80^.

## Supplementary Information

Supplementary Information.

## Data Availability

Data for all variables used in the statistical analyses will be made available on request. Extensive sets of uncompressed raw video RGB data, Kinect data, load platform voltage data and kinematic x,y,z data are also available on reasonable request.
