# Supplementary material for: Quantifying defence cascade responses as indicators of pig affect and welfare using computer vision methods
Source: Sci Rep. 2020 Jun 2;10:8933. doi: 10.1038/s41598-020-65954-6 (PMC7265448; doi:10.1038/s41598-020-65954-6)
Supplement: Supplementary file 1 — Supplementary Information. [file 41598_2020_65954_MOESM1_ESM.docx]

**Quantifying defence cascade responses as indicators of pig affect and welfare using computer vision methods**

**Poppy Statham, Sion Hannuna, Samantha Jones, Neill Campbell, G. Robert Colborne, William J. Browne, Elizabeth S. Paul & Michael Mendl**

**Supplementary Information**

**Materials and Methods**

***Test set up***

See Fig. S1.

***Synchronisation of load platform, video, Kinect and Kinematic data***

Load cell voltages from the force measuring pen were digitized into 14bit values using an Advantech- USB-4704 (48 kS/s, 8-channel) Portable Data Acquisition Module and synchronised data from the load platform, fast-capture video cameras, and Kinect cameras were fed to a quad core i7 computer (Computer 1) with 3 firewire 800 capture cards and 4 x 160GB Western Digital Velociraptor hard disk drives in a RAID0 array for storage and subsequent analysis. Kinematic data from the four Qualisys infrared cameras were converted into digital form using a bespoke A/D board and stored on a separate Dell computer (Computer 2). Computer 2 also received synchronised load platform data, allowing temporal alignment of the Kinematic data with the video and force data. Specifically, the temporal offset which maximised the normalised cross-correlation for the force data captured by the two computers was exploited for alignment. On Computer 1, force data were captured at the maximum rate (400Hz) by a dedicated process. The latest value was stored in a shared memory location, which was copied by the Kinect capture process and video every time they had grabbed a sample (3 video frames for the cameras and 1 RGB and 1 depth image for the Kinect). This ensured that the force data were properly synchronised with the other data at time of capture. These values were also time stamped to verify the capture had gone according to plan in post processing. The force values used in analyses were those captured alongside the video data on Computer 1.

***Justification for using individual KLT points***

Rather than finding the centroid of the KLT points and then differentiating and double differentiating its position across sequential frames to calculate velocity and acceleration respectively, we instead extracted the magnitude of velocities and accelerations for each point individually and then took the average of the magnitude of those points' trajectories. This eliminated situations where motions in opposite directions would cancel each other out when taking averages. Consider the situation where a pig splays its legs when startled to consolidate its base of support. Its centre of mass may move vertically down but not be displaced horizontally and hence not appear to change from an overhead view. However, there is certainly a reaction to the stimulus and considering individual points (e.g. on limbs), rather than estimating a centroid, facilitates recording this reaction.

***Use of acceleration of 50 KLT points for the KLT Startle Magnitude analyses***

The raw output of the KLT tracker is a set of horizontal and vertical image coordinates for each KLT point per frame. The change in coordinates of a particular feature from one frame to the next is the velocity in pixels per frame for that feature. The difference in velocities between subsequent frames for a given feature is acceleration in pixels per frame squared. As we only consider the magnitude of these measures, we refer to them as speed and acceleration respectively. Acceleration is closely related to force, and the time window for measuring maximum acceleration was empirically determined by watching videos of the pigs, alongside acceleration traces, and identifying the time by which all had reached peak acceleration. *x* KLT points were analysed per frame, with data being extracted for a range of *x* values (from 10 to 150) in order to determine whether the number of KLT points affected results. KLT measures using 50 points yielded the highest average correlation coefficient and were therefore used in all analyses (Fig. S2). Acceleration of KLT points across adjacent frames was calculated as the mean acceleration for the 50 KLT points displaying the largest accelerations. A by-eye check showed that at least 50 points were tracking the pig in all videos and 50 points were ample to average out Gaussian noise for tracked points. The peak absolute values of these mean accelerations across the 0.7 s time window was, therefore, the *KLT Acceleration Startle Magnitude*.

***Freeze threshold calculations***

Freeze thresholds were calculated using 10-fold cross validation. The image analysis (IA) data set was split into 90% training and 10% testing data. The training data were used to determine a threshold as follows. A single threshold was selected by performing an exhaustive search of the range of values the thresholds might reasonably take. Specifically, the range for each of the measures was divided into 100 discreet steps. Next the total error (the sum difference between hand-labelled freeze durations and the ones extracted from the IA time series) was calculated for each of these 100 thresholds for each of three measures (KLT speed, KLT acceleration, load platform reading). The threshold selected for each measure was the one that produced the lowest total error for that measure. The three thresholds for each of the measures were then used to calculate the freeze durations for the test data. This process was repeated 10 times until freeze durations were available for all test sequences. Freeze durations were calculated using the force data, the KLT point speed and acceleration and the Kinect data speed and acceleration.

***Kinect data time series construction***

For the overhead Kinect depth map sequences, the silhouettes defining the pigs' bodies were deduced using a combination of thresholding and connected component analysis. Once silhouettes had been extracted for each frame, the centre of mass was simply the mean of the depth pixels defining the pig. Because the depth axis was measured in real world units (distance from the Kinect sensor in mm) and the other two axes in the image plane were measured in pixels, it was not meaningful to calculate 3D trajectories without further post processing.

**Supplementary Tables and Figures**

**Table S1.** Rating scale used to describe ‘relaxed-tense’ state of pigs on the load platform prior to the first test in each session.

| **Score** | **Behaviour description** |
| --- | --- |
| 1 | Completely calm, relaxed and behaving normally |
| 2 | Slightly tense and more aware of surroundings |
| 3 | Moderately tense, eating food treats but alert |
| 4 | Tense, not eating food treats, constantly alert |
| 5 | Very tense, trying to find escape route throughout |

**Table S2.** Ethogram of behaviour patterns recorded during test sessions. Events and States occurred during the Defence Cascade response and Outcomes terminated the response.

| **Behaviour** | **Behaviour Type** | **Description** |
| --- | --- | --- |
| Jump and fall | Event | Jump in the air in response to stimulus, knees or abdomen touch the ground on landing |
| Jump away | Event | Jump in response to stimulus, and away from it |
| Jump on spot | Event | Jump in response to stimulus and land standing in same location |
| Spin to face | Event | Whole body movement to re-orientate towards stimulus |
| Side step | Event | One sideways movement with one or two feet, typically away from the stimulus |
| Head up | Event | Lift head suddenly in response to stimulus, snout no longer in close contact with floor |
| Head turn | Event | Head turn in the direction of the stimulus |
| Muscle ripple | Event | Tensing of muscles or muscle twitch in response to stimulus |
| Ear prick | Event | Ears pulled back or pointing upwards in response to stimulus |
| Freeze | State | Muscles tensed, whole body stationary, ears often pricked |
| Flee | Outcome | Rapid exit from the force pen in response to stimulus |
| Slowly leave | Outcome | Slow, controlled/relaxed exit from force pen (muscles not tensed, ears not pricked) |
| Return to normal behaviour | Outcome | Return to exploratory or food related behaviours whilst exhibiting a relaxed posture. |

**Table S3**. Factors analysed to determine their influence on observer startle and freeze measures.

| **Factor** | **Categories / Range** | **Reason for analysing this factor** |
| --- | --- | --- |
| Sex of pig | Female / Male | Pig sex may influence startle and freeze responses |
| Weight of pig | 23-78 (kg) | Pig weight may influence the magnitude of a startle response |
| Stimulus used | Balloon / Balloon & Real Person / Model Person / Umbrella / Real Person / Bucket / Bin Bag | Startle and freeze responses may vary depending on stimuli and their auditory / visual impact |
| ‘Relaxed-tense’ score | 1-5 | The degree of behavioural relaxation prior to a test may influence startle and freeze responses |
| Behaviour when stimulus delivered | Food related / Alert / Door Directed Behaviour / Pen Explore / Enrichment explore | Behaviour at the time of stimulus delivery may affect startle and freeze responses |
| Orientation | Back to stimuli / 90 degrees / Facing stimuli | Whether the stimulus is within the pig’s field of vision may affect startle and freeze responses |
| Test type | Standard or Kinematic | Test type reflects the amount of prior handling which may, in turn, influence startle and freeze responses |
| Day of test | 1-72 | Indicates the day on which the test took place, some factors e.g. weather, may vary between different test days and influence startle and freeze responses |
| Week of experiment | 1-6 | Indicative of the age of pigs which may influence startle and freeze responses |
| Session | 1-8 | Indicates number of tests pigs have undergone which may influence startle and freeze responses |
| Test Number | 1-5 | Startle and freeze responses may vary across repeated tests within a session |
| Start time | 09:00 – 18:00 | Time of day and circadian rhythms may influence startle and freeze responses |

**Figure S1.** Pig’s eye view of the force-measuring pen and load platform through the entrance door. The photograph shows two fast-capture video cameras, one positioned to the side of the pen and one positioned overhead alongside a Microsoft Kinect depth camera, a standard video camera positioned to the side of the pen, and four infrared kinematic cameras which were positioned at the four corners of the pen during weeks 4 and 11 of data collection (photograph by Tracy Townsend).


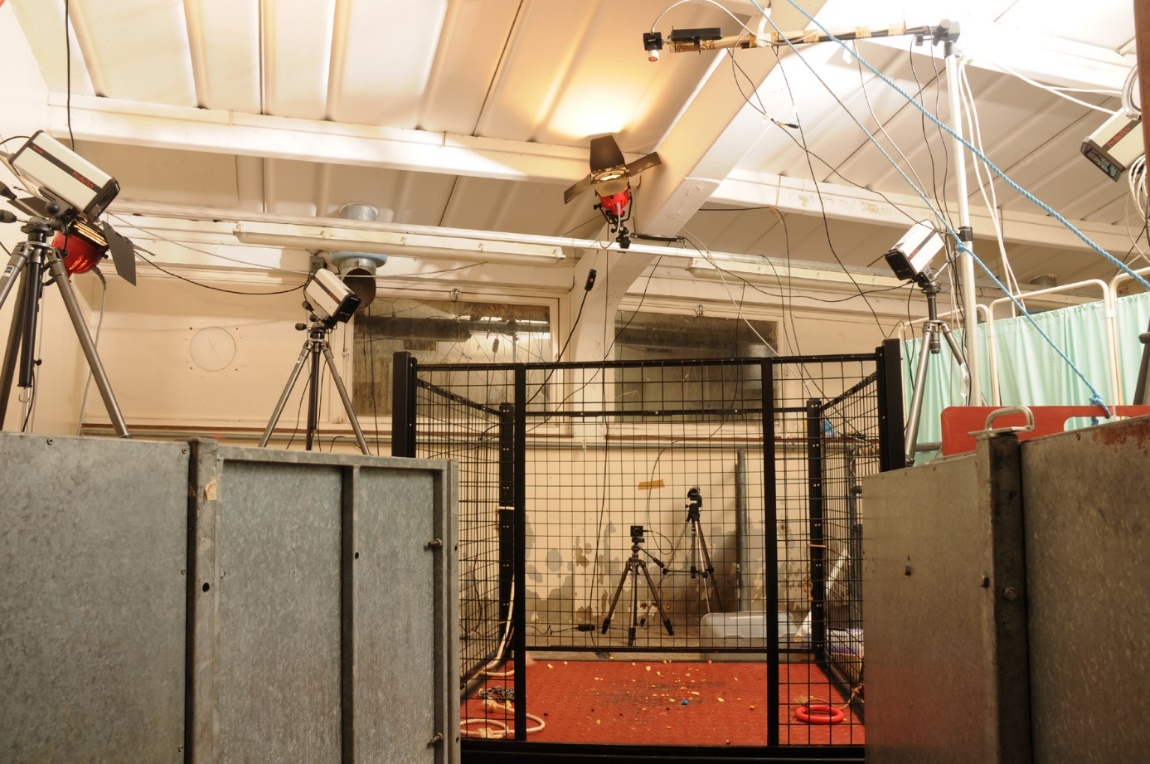


**Figure S2**. KLT acceleration measures of startle magnitude using different numbers of KLT points (10-150) were correlated with all the other measures of startle magnitude to determine which number of points generated the best correlation. The figure shows the resulting correlation coefficients.
